# Supplementary material for: Metagenomic Analysis of the Composition of Microbial Consortia Involved in Spruce Degradation over Time in Białowieża Natural Forest
Source: Biomolecules. 2023 Sep 28;13(10):1466. doi: 10.3390/biom13101466 (PMC10604581; doi:10.3390/biom13101466)
Supplement: Supplementary file 1 [file biomolecules-13-01466-s001.zip › Figure S1.pdf]

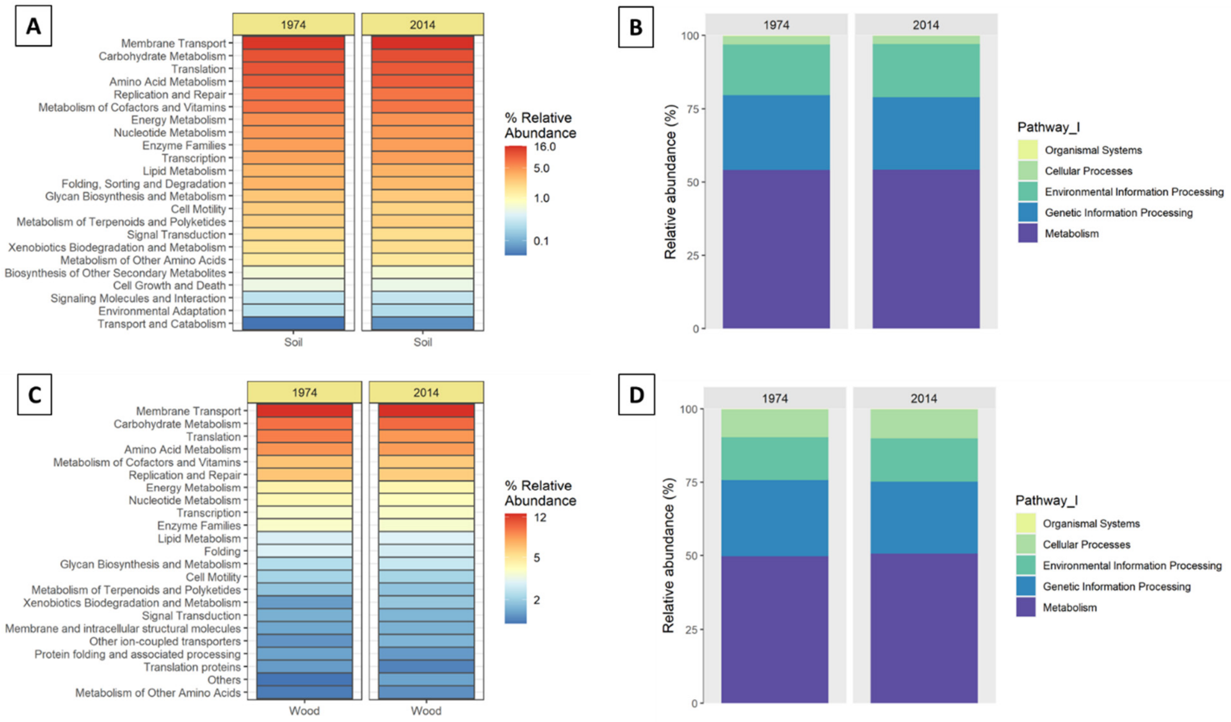

Supplementary Figure S1. Functional profiling of bacterial communities of wood and soil performed with PICRUSt.
