# Supplementary material for: Metagenomic Analysis of the Composition of Microbial Consortia Involved in Spruce Degradation over Time in Białowieża Natural Forest
Source: Biomolecules. 2023 Sep 28;13(10):1466. doi: 10.3390/biom13101466 (PMC10604581; doi:10.3390/biom13101466)
Supplement: Supplementary file 1 [file biomolecules-13-01466-s001.zip › Tables S1-S3.pdf]

Supplementary Table S1. FASTQ Reads and merging statistics

No	Sample name	sample type	Read Pairs obtained	Yield (Kbp)	%Q30	Mean Q	Percent of PE combined	Mean of lengths	effective tags (after preprocessing and chimera removal)
**wood and soil samples 1974**									
1.	V3V4a	soil 1974_1	105266	59784	79.09	33.14	96.79%	406	104836
2.	V3V4a	wood 1974_1	90669	51494	79.26	33.18	97.38%	408	90482
3.	V3V4a	soil 1974_2	95534	54267	80.10	33.37	98.02%	407	94570
4.	V3V4a	wood 1974_2	99083	56278	79.62	33.26	97.66%	407	98578
5.	V3V4a	soil 1974_3	94544	53701	80.10	33.38	97.95%	407	94062
6.	V3V4a	wood 1974_3	107894	61275	79.86	33.31	97.59%	407	107456
7.	ITS1b	soil 1974_1	108670	54887	76.78	32.50	95.74%	245	108536
8.	ITS1b	wood 1974_1	115504	58544	76.09	32.31	94.39%	255	115123
9.	ITS1b	soil 1974_2	84392	44547	76.45	32.46	95.77%	261	84086
10.	ITS1b	wood 1974_2	96497	48713	82.36	33.75	97.68%	253	96446
11.	ITS1b	soil 1974_3	155775	79274	77.79	32.77	96.20%	246	155638
12.	ITS1b	wood 1974_3	89158	46042	79.53	33.14	98.54%	256	88681
**wood and soil samples 2014**									
13.	V3V4a	soil 2014_1	128530	72998	68.32	30.51	89.38%	407	112718
14.	V3V4a	wood 2014_1	102880	58445	69.05	30.68	90.51%	406	109020
15.	V3V4a	soil 2014_2	125628	71354	68.41	30.53	90.31%	406	131975
16.	V3V4a	wood 2014_2	113071	64234	68.91	30.64	90.03%	406	127608
17.	V3V4a	soil 2014_3	109468	62178	68.93	30.66	90.61%	406	102667
18.	V3V4a	wood 2014_3	132519	75282	69.96	30.91	92.08%	406	125118
19.	ITS1b	soil 2014_1	115567	57843	83.25	34.01	97.58%	244	115340
20.	ITS1b	wood 2014_1	91870	50230	84.61	34.34	98.23%	273	91771
21.	ITS1b	soil 2014_2	129837	67340	82.02	33.77	98.30%	257	129471
22.	ITS1b	wood 2014_2	111359	61317	80.29	33.38	97.51%	279	111233

23.	ITS1b	soil 2014_3	99703	52735	79.18	33.06	98.34%	289	99555
24.	ITS1b	wood 2014_3	106567	57680	81.98	33.75	97.18%	270	106300

Supplementary Table S2. Comparison of alpha diversity of bacterial microbiome of wood logs fallen in 1974 and 2014 and soil from under the logs based on Chao1, Shannon index and Simpson diversity index.

	**Chao1**	**Shannon index**	**Simpson diversity index**
**wood**			
**1974_1_rep**	56	2.896	0.922
**1974_2_rep**	67	2.864	0.91
**1974_3_rep**	69	3.039	0.918
**2014_1_rep**	57	2.895	0.897
**2014_2_rep**	59	3.177	0.919
**2014_3_rep**	60	2.961	0.908
**soil**			
**1974_1_rep**	59	2.798	0.905
**1974_2_rep**	72	2.968	0.918
**1974_3_rep**	48	2.671	0.887
**2014_1_rep**	80	3.296	0.944
**2014_2_rep**	44	2.718	0.908
**2014_3_rep**	66	3.177	0.932

Supplementary Table S3. Comparison of alpha diversity of fungal microbiome of wood logs fallen in 1974 and 2014 and soil from under the logs based on Chao1, Shannon index and Simpson diversity index.

	**Chao1**	**Shannon index**	**Simpson diversity index**
**wood**			
**1974_1_rep**	112	3.206	0.924
**1974_2_rep**	96	2.415	0.803
**1974_3_rep**	71	2.531	0.843
**2014_1_rep**	40	1.379	0.59
**2014_2_rep**	44	1.073	0.381
**2014_3_rep**	50	2.104	0.786
**soil**			
**1974_1_rep**	104	3,169	0,907
**1974_2_rep**	73	2,452	0,833
**1974_3_rep**	90	1,995	0,686
**2014_1_rep**	103	3,212	0,921
**2014_2_rep**	102	2,404	0,819
**2014_3_rep**	109	2,598	0,813
